# A mating method accounting for inbreeding and multi-trait selection in dairy cattle populations

**DOI:** 10.1186/1297-9686-41-7

**Published:** 2009-01-05

**Authors:** Jean-Jacques Colleau, Kevin Tual, Hervé de Preaumont, Didier Regaldo

**Affiliations:** 1 INRA, UR337 Station de génétique quantitative et appliquée 78352 Jouy-en-Josas Cedex, France; 2 Centre d'Insémination Animale, BP54, 61382 L'AIGLE Cedex, France; 3 Institut de l'élevage, Département Génétique, 149 Rue de Bercy, 75595 Paris Cedex 12, France

## Abstract

Selection in dairy cattle populations usually takes into account both the breed profiles for many traits and their overall estimated breeding values (EBV). This can result in effective contributions of breeding animals departing substantially from contributions optimised for saving future genetic variability. In this work, we propose a mating method that considers not only inbreeding but also the detailed EBV of progeny or the EBV of sires in reference to acceptance thresholds. Penalties were defined for inbreeding and for inadequate EBV profiles. Relative reductions of penalties yielded by any mating design were expressed on a scale ranging from 0 to 1. A value of 0 represented the average performance of random matings and a value of 1 represented the maximal reduction allowed by a specialized, single-penalty, mating design. The core of the method was an adaptative simulated annealing, where the maximized function was the average of both ratios, under the constraints that both relative penalty reductions should be equal and that the within-herd concentration criterion should be equal to a predefined reasonable value. The method was tested on two French dairy cattle populations originating from the same AI organization. The optimised mating design allowed substantial reductions of penalty: 70% and 64% for the Holstein and the Normandy populations, respectively. Thus, this mating method decreased inbreeding and met various demands from breeders.

## Introduction

Over time, management of genetic variability and avoidance of inbreeding have become major issues in dairy cattle selection. Currently, yearly inbreeding rates in French dairy cattle breeds range between 0.09–0.22% [1] and consequently, it can be assumed that in the next two to three decades inbreeding coefficients will become very substantial and very likely, harmful to the fitness of these populations. Extensive quantitative genetic studies have been carried out on how to manage genetic variability and contain inbreeding while selecting for economically important traits. In most cases, it has been proposed to constrain inbreeding rates to desired values (typically 1% per generation) and then to maximize genetic gain through optimised contributions of breeding animals [2,3]. Given that genetic gain has been modelled for one trait, this theory is relevant to selection on a single trait or on a fixed linear combination of traits *i.e. *an overall selection objective.

However, as reported worldwide, selection strategies in dairy cattle have chosen breeding animals not only with high overall Estimated Breeding Values (EBV) but also without major faults for the detailed traits. In the past, breeders have worked mainly on production traits, milk content traits and type traits. However, today, functional traits are more and more taken into consideration, especially mastitis traits (cell counts and cases of clinical mastitis), fertility and longevity traits because of unfavourable consequences of selection during the last decades [4]. In this context, breeding animals with rare profiles have become popular and used extensively, which has definitely contributed to inflate inbreeding rates. In addition, it is anticipated that breeders may exclude some breeding animals on account of faults although these animals should be saved for genetic conservation.

To avoid this situation, an optimised mating plan can be proposed where mating some sires to females with excellent corresponding traits would minimize these sire's faults. Mate selection [5-7] has been used to optimise the expected value of progeny, mixing the different issues of selection and mating design. Sonesson and Meuwissen [8] have proposed an optimised mating plan considering only known sire contributions (maximizing the expected genetic gain under the constraint of a desired inbreeding rate) but not detailed EBV (single-trait context).

The objective of the present paper was to examine the potential of a two-step approach in a multi-trait context in order to contain inbreeding development while producing a progeny with a profile likely to be accepted. First, we describe the analytical method in detail and second, we present the results of a test on two French dairy cattle populations for which requests of breeders for individual cows were often known from routine surveys.

## Methods

### The analytical method

#### General principles

A two-step approach was used. Contributions of sires and then those of matings were optimised through the same stochastic method *i.e.*, an adaptative simulated annealing (ASA) that maximizes a leading function penalized for constraints (see Appendices 1 and 2). In the first step, alternative solutions for the ASA process were obtained by exchanging the fate of a randomly chosen pair of used-unused available semen doses (see Appendix 3). In the second step, sires attributed to a pair of randomly chosen dams were permuted [8].

#### Constraints for optimising contributions of selected sires

The problem is similar to that addressed analytically by [9], except that in our case, constraints on available semen doses had to be accounted for. Given a desired average overall EBV, $\tilde{W}$, for selected sires, contributions were calculated so as to minimize the average coancestry coefficient $\bar{\varphi }$ in the existing female population and augmented by that of the future females resulting from the proposed matings. Thus, the leading function was $-\bar{\varphi }(k)$ and the penalizing function was ${\left(\bar{W}(k)-\tilde{W}\right)}^{2}$ for configuration *k *of sire contributions. The details of the ASA for finding the optimal contributions given the constraints on doses and on the average overall EBV of selected sires are shown in Appendix 3.

#### Penalty components for optimising matings

When optimising matings, the leading function and constraints accounted for several penalty components. The first component, $\bar{F}$(*k*), is simply the average inbreeding coefficient of matings for the current configuration being tested, denoted *k*. The second component, $\bar{T}$(*k*), is the average penalty of current matings for traits. The T-penalty for an individual mating considers the EBV for some traits, in comparison with desirable values where the desirability function might be cow-dependent. Basically, two major cases should be considered. In case 1, the owner of the cow does not express specific requests for this cow and in case 2, he explicitly requests that the sire chosen for this cow has a high EBV for some traits elected within a very wide list (production, type and functional traits, and even an overall objective). To address case 1, the breeding organization chose a list of traits for which thresholds were defined and the value of a mating was assessed by the number of faults D, *i.e*., the number of traits for which the expected EBV of progeny was below these thresholds. In other words, the breeding organization made the assumption that breeders would exclude matings with EBV too unfavourable for progeny. To define the T-penalty, the obvious heterogeneity of requests (between cases and within case 2) must be circumvented by using a homogeneous penalty system, otherwise, during the ASA process, more attention will be paid to matings involving cows with more variable T-penalties, inducing an involuntary preferential treatment of these cows. Thus, the penalty system was standardized for variances. For case 1, the T-penalty for mating *ij *(cow *i *mated to sire *j*) is defined by $T_{ij}=\frac{D_{ij}-{\left(D_{ij}\right)}_{\min}}{\sigma (D_{ij})}$ where the minimum and the standard deviation were obtained by considering the whole mating set (number of matings = number of sires * number of females). For case 2, we considered the cow-dependent request function B (B for breeder) of the sire's EBV for traits, defined by its owner, based on a single trait or a linear combination of traits. After standardization, the penalty is defined by $T_{ij}=\frac{{\left(B_{s}^{\left[i\right]}\right)}_{\max}-B_{j}^{\left[i\right]}}{\sigma (B_{s}^{\left[i\right]})}$ where the maximum and the standard deviation were obtained by considering the whole set of sires (*s *= 1 to the number of sires). Thus, the T-penalties were adjusted so that the best mating considered was not penalized at all. Consequently, these penalties were standardized for variances only and not for expectations that depended on the cows. However, it can be easily shown that differences of expectations have strictly no effect on the SA process.

The last component of the penalty function was *C*(*k*), the within-herd concentration of recommended sires, because recommending too few sires in the same herd might motivate breeders to partly use other sires. Let *h *be the herd index, *j *the sire index, *N*_*h *_the number of cows in herd *h*, *N*_*hj *_the number of cows to be served by sire *j *in herd *h *. The concentration penalty used during the optimisation procedure is $C=\sum_{h}\sum_{j}N_{hj}^{2}$. Minimizing this criterion would minimize the within-herd variance of the numbers of sire recommendations (including 0's), because for each herd, the sum of recommendations is equal to the number of cows. C was constrained to be equal to a desired value $\tilde{C}$, according to the tolerance of breeders. Of course, breeders might be more or less tolerant according to country, area, breed and so on. In the test example, practitioners required that for very large herds, the fraction of cows to be inseminated by the same bull did not exceed 10%. This breeder-defined criterion of concentration should be adjusted according to herd size, especially when considering small herds, where this criterion would be automatically in excess even when each cow was mated to different bulls. Thus, the maximal number of cows mated to the same bull in the same herd was defined by *M*_*h *_= *ceiling*(0.1*N*_*h*_), the closest integer larger than or equal to 0.1*N*_*h*_, i.e., 1 for sizes 1–10, 2 for sizes 11–20, ..., 10 for sizes 91–100 and so on. Finally, the analytical C-penalty was translated into a simple field concentration indicator *i.e. *the fraction of cows located in herds where one or several sires were recommended too frequently. Choosing this fraction also depended on the tolerance of breeders. In the test examples, practitioners considered that this fraction should not exceed 0.10. The SA procedure for reducing C-penalty alone was run until the field indicator, calculated at the end of each temperature step, met this requirement. Then, the desired value $\tilde{C}$ was the last *C *obtained.

#### Functions involved when optimising matings

Let us define the efficiency ratios, lying between 0 and 1, $\rho_{F}(k)=\frac{\bar{F}(0)-\bar{F}(k)}{\bar{F}(0)-\min_{k}\bar{F}(k)}$ and $\rho_{T}(k)=\frac{\bar{T}(0)-\bar{T}(k)}{\bar{T}(0)-\min_{k}\bar{T}(k)}$ where configuration 0 corresponds to the expectation of configurations under random matings and where the minima are obtained after specific minimizations for $\bar{F}$ and $\bar{T}$ separately. These ratios correspond to the relative penalty reductions yielded by the optimised mating design after starting from random matings. Obviously, the goal of the optimisation was to increase these ratios. In addition, the full balance between these increases was requested because usually breeders have demands on many aspects at the same time. Then, the leading function is $\rho (k)=\frac{\rho_{F}(k)+\rho_{T}(k)}{2}$ subject to the constraint function (*ρ*_*F*_(*k*) - *ρ*_*T*_(*k*))^2 ^and also to the constraint function (*C*(*k*) - $\tilde{C}$)^2^, as stated earlier. The values obtained for *ρ *were observed every tenth temperature and ASA was stopped when both relative increases (compared with the averages obtained during the ten previous temperatures) were lower than 0.05%.

#### Simulated annealing accounting for forbidden matings

Some matings were forbidden for reasons not addressed in the penalty function, *e.g.*, expected calving difficulties given the corresponding EBV of the sire and the heifer status of the females or a substantial risk for transmitting a genetic defect. Matings with high penalties for F or T were also forbidden, because likely to be strongly rejected by breeders. Using dummy deterring penalty values for these matings might disturb the adaptative simulated annealing. Thus, the following two-step procedure was implemented. First, a SA decreasing the number of forbidden matings retained in the current solution was run to obtain a completely allowed mating design (about 30 runs of N permutations). Second, starting from this mating design, the ASA was implemented on allowed permutations.

Finally, the full method led to five optimisations (four SA and one ASA). The first one provided an initial mating plan free from forbidden matings. The second and the third ones provided the minimal penalties $\bar{F}$ or $\bar{T}$, respectively. The fourth one provided the value of the concentration penalty to be considered as a constraint. The fifth one was the adaptative simulated annealing, constrained according to the results of previous procedures.

### Material

Data originated from the AI cooperative of L'Aigle (Normandy, France) and included 151179 heifers and cows (89483 Holstein and 60696 Normandy) likely to be inseminated by selected AI sires (excluding young sires) between August 1, 2007 and July 30, 2008. Mating recommendations obtained from these data were sent to breeders in July 2007 (more recent data, not exploited in the present paper, were used for recommendations sent in July 2008).

Animals were located in 2094 herds (average size 72, standard deviation 32). About half of the herds (1114) exploited both breeds, a situation frequently met in the area of the Cooperative. After splitting the data according to breeds, the numbers of herds considered were 1725 and 1483 for the Holstein and the Normandy breeds, respectively.

The number of candidates for service was 24 in Holstein and 28 in Normandy. The available semen corresponded to 117% and 129% of the needs (1.8 and 1.6 dose per gestation in Holstein and Normandy). The major requirement for calculating the numbers of females served by the selected candidates was that their weighted average overall EBV (ISU) (2007 evaluation rolling basis) should be the same as the average observed on the inseminations carried out in 2006–2007 (2006 evaluation rolling basis), *i.e.*, 147 for the Holstein breed and 130 for the Normandy breed. Then, selection pressure after progeny-testing was maintained constant over the period 2006–2008. As a result, 24 Holstein sires and 26 Normandy sires were retained.

The Cooperative has been proposing mating plans to its breeders for many years, with the possibility to indicate the desired EBV profiles for the sires to be mated to their individual cows. The answers obtained at the beginning of the year 2007 for the females previously mentioned were included in the data set analysed. The breeders were allowed to choose up to three traits according to the cow, from a list of 33 (Holstein) or 30 (Normandy) traits and to give their priority in case of multiple requests. The majority of the traits proposed were type traits (21 and 19, respectively) followed by functional traits (seven and six) and production traits (five and five). Details are indicated in Appendix 4. In order to calculate the number of faults, simplified EBV of the progeny (0.5 sire EBV +0.25 maternal grand-sire EBV) were considered for 12 traits in both breeds in reference to thresholds also indicated in Appendix 4 (six for type, three for functionality, three for production in the Holstein breed, and seven for type, three for functionality and two for production in the Normandy breed). The breeder function mentioned previously was a linear function of standardized sire EBV (after dividing by the corresponding genetic standard deviation). When requesting two traits, weights of traits 1 and 2 were 1 and 0.67 respectively, and when requesting three traits, the coefficients were 1, 0.6 and 0.4.

Herds could be split into herd group 1 (non-returning requests) and herd group 2 (returning requests). The proportions of herds 2 were 32% and 47% in Holstein and Normandy, respectively, *i.e.*, corresponding to strong minorities. The average size of Holstein herds 2 was substantially smaller than that of herds 1 (43.4 *vs. *57.7), a phenomenon not observed in the Normandy breed (42.1 *vs. *39.9).

The lists of proposed traits were used extensively because each trait was mentioned at least once. This resulted in an extremely heterogeneous demand: as many as 3656 and 3485 distinct profiles were mentioned, in Holstein and Normandy, respectively. Fifty percent of the cows were concentrated on 75 and 87 profiles according to the breed whereas 10% of the cows were dispersed over 2225 and 2107 profiles. Analysing the demand in the Holstein breed, after weighting for the number of involved cows, requests for one, two and three traits were 24%, 32% and 44%, respectively. The most mentioned trait was a production trait (43%), followed by a type trait (40%) and a functional trait (17%). In the Normandy breed, requests for one, two and three traits were 20%, 28% and 52%, a situation similar to that in the Holstein breed (breeders were prone to mention trait triplets). The first trait mentioned concerned type (55%) followed by production (34%) and functionality (11%). Finally, in both breeds, breeders paid much attention to type and production traits and included functionality mainly in combination with other traits.

Sires with an EBV for ease of birth, lower or equal to 87 in Holstein and 86 in Normandy, were forbidden for heifers, which led to forbidding respectively 12.9% and 7.5% of all the matings. In the Holstein breed, sire carriers of the CVM (complex vertebral malformation) abnormality were forbidden for the daughters of carriers, which increased to 15.6% of the proportion of forbidden matings. Extreme inbreeding coefficients (higher than 8.5% and 8%, respectively) were also forbidden, resulting in overall forbidding rates of 17.2% and 14.2%. These rates increased to 26.4% and 21% after forbidding matings that exhibited more than four faults, even in herds 2. Final forbidding rates amounted to 37.1% and 37.5% after excluding in herds 2, the matings being more penalized than the average within-cow penalty. As a result, the forbidding rates were much stronger in herds 2 (60.1% and 56% according to the breed) than in herds 1 (26.4% and 21%).

## Results

### Optimised penalty reductions in the current situation

Constraints found for the concentration penalty in both breeds were about halfway between the concentration penalty obtained with random mating and the minimum penalty (*ρ*_*c *_= 0.5). Table 1 shows that specific optimisations could produce substantial responses. The average T-penalty could be reduced by about 50–60% in both breeds, in comparison to the average T-penalty of the allowed (already highly selected) matings, a fact likely to interest breeders. Relative responses on the average inbreeding coefficient were also substantial (minus 25–30%). The next step was to examine whether the optimisation method proposed could recover a significant part of this potential. Optimisation was conducted over 230 temperatures for the Holstein breed and 270 temperatures for the Normandy breed, constraining the relative reductions for the F-penalty and the T-penalty to be the same. Table 1 shows that this requirement was fulfilled. For these penalties, relative penalty reductions were about 70% in Holstein and 64% in Normandy. Therefore, optimisation could be considered as fairly efficient and an acceptable trade-off between the three conflicting penalties was found.

**Table 1:** Effect of the optimized mating method on inbreeding coefficients (F), trait penalties (T) and sire concentration within herd (C)

Mating method	Holstein breed allowed: 62.9%			Normandy breed allowed: 62.5%		
	F (%)	T	C/1000	F (%)	T	C/1000

Random	3.82	2.16	399	4.05	1.50	240

Specific	2.91	1.10	311	2.80	0.58	179

Optimized *ρ *(%)	3.18 69.8	1.42 69.8	355 50.0	3.25 63.9	0.91 63.9	210 50.0

*ρ *= relative penalty reduction

### Detailed results obtained for the current situation

Table 2 presents the detailed results obtained for the Holstein breed. In herds 1, optimisation made it possible to decrease the inbreeding coefficient by about 1% (25% of the value with random matings) and the numbers of faults by about 1 (30% of the value with random matings). Optimisation also reduced the standard deviations and the maxima. Only 39 herds (mostly with a size smaller than 30) out of 1069 could be considered as presenting an excessive use of some bulls. In herds 2, the initial inbreeding coefficient was higher than in herds 1 (+0.3%), which was apparently due to longer pedigrees of dams (7.0 *v*s. 6.4 generations) and may be the consequence of older herd involvement in recording and breeding schemes. This fact indicated that the higher average inbreeding of the optimised matings in herds 2 was not the direct consequence of breeders' requests. Optimisation was almost ineffective in herds 2 on the number of faults but succeeded in reducing by half the average T-penalty as expected. Unsurprisingly, a significant proportion of herds 2 (205/606), mostly with a size smaller than 50, exhibited concentration problems. Hopefully, this kind of problem might be more accepted by breeders of herds 2 because they have strong requirements for bulls.

**Table 2:** Detailed results in the Holstein breed

Mating method		Herds 1 (without requests) average/sd/min/max	Herds 2 (with requests) average/sd/min/max
Random (any mating)	Inbreeding (%) Faults T-penalty	3.95/2.13/0/30.86 2.94/1.19/0/9 2.47/1.00/0/7.57	4.26/1.94/0/30.12 2.91/1.18/0/9 2.09/1.00/0/6.72
			
Optimized (allowed)	Inbreeding (%) Faults T-penalty	3.02/1.30/0/6.74 2.04/0.99/0/4 1.71/0.83/0/3.36	3.37/1.19/0/7.35 2.70/0.99/0/4 0.94/0.78/0/3.31

T-penalty = penalty for traits

Table 3 presents the Normandy version of Table 2. In herds 1, optimisation made it possible to decrease the inbreeding coefficient by about 1.4% (30% of the value with random mating) and the numbers of faults by about 0.9 (40% of the value with random matings). Only 32 herds (mostly with a size smaller than 30) out of 805 could be considered as presenting an excessive use of some bulls, a result analogous to that obtained in Holstein. In herds 2, the initial inbreeding coefficient was higher than in herds 1 (+0.4%), exactly like in the Holstein breed, due to longer pedigrees of dams (7.8 *vs. *7.1 generations) and likewise, this fact mostly explained why the average inbreeding of the optimised matings was higher in herds 2 than in herds 1. Like in Holstein, optimisation was almost ineffective for the number of faults but succeeded in reducing by half the average T-penalty. A significant proportion of herds 2 (193/678) presented concentration problems, as in Holstein.

**Table 3:** Detailed results in the Normandy breed

Mating method		Herds 1 (without requests)	Herds 2 (with requests)
		**Average/sd/min/max**	**Average/sd/min/max**

Random (any mating)	Inbreeding (%) Faults T-penalty	4.43/2.99/0/32.48 2.23/1.41/0/8 1.58/1.00/0/5.69	4.85 2.830 33.96 2.18/1.40/0/7 2.04/1.00/0/5.59
			
Optimized (allowed)	Inbreeding (%) Faults T-penalty	3.04/1.38/0/7.72 1.31/1.06/0/4 0.93/0.75/0/2.83	3.51/1.25/0/7.98 2.11/1.25/0/4 0.89/0.74/0/3.06

T-penalty = penalty for traits

### Effect of absence of forbidding on F and T penalties

The results are shown in Table 4 and its comparison with Table 1 obviously shows that forbidding some matings, especially based on T penalties, prevented us from finding better solutions for the average inbreeding coefficients (by about 0.1% in terms of probability) and conversely, generated lower values for T-penalties (by 0.04). Thus, the forbidding system was not neutral towards penalties *i.e. *it clearly favoured the reduction of T-penalties, although by a reasonable amount.

**Table 4:** Efficiency of the mating method for reducing inbreeding coefficients (F), trait penalties (T), *without forbidding for F and T*

Mating method	Holstein breed allowed: 84.5%		Normandy breed allowed: 85.3%	
	F (%)	T	F (%)	T

Random	3.89	2.39	4.05	1.83

Specific	2.86	1.06	2.73	0.56

Optimized *ρ *(%)	3.14 72.8	1.28 72.7	3.12 70.5	0.94 70.5

*ρ *= relative penalty reduction

### Effect of other scenarios

Table 5 shows that, without any request on traits, a lower inbreeding coefficient could have been obtained as expected. Conversely, if requests had been expressed by all the breeders and had even focused on the most frequently demanded profiles, inbreeding would have been higher, but by a moderate amount. Consequently, it was concluded that the method was rather robust to multiple demands for detailed traits.

**Table 5:** Efficiency of the mating method for reducing inbreeding coefficients (F), trait penalties (T) in the Holstein (*Normandy*) breed, using three scenarios

Mating method	32% herds 2 *47% herds 2* actual allowed: 62.9% *allowed: 62.5%*		0% herds 2 *0% herds 2* no profiles allowed: 73.6% *allowed: 79%*		100% herds 2 *100% herds 2* profiles 50% allowed: 35.8% *allowed: 39.3%*	
	F (%)	T	F (%)	T	F (%)	T

Random	3.82 *4.05*	2.16 *1.50*	3.85 *4.10*	2.33 *1.51*	3.85 *4.10*	1.32 *1.38*

Specific	2.91 *2.80*	1.10 *0.58*	2.88 *2.74*	1.43 *0.78*	3.05 *2.90*	0.83 *0.90*

Optimized *ρ *(%)	3.19 *3.25* 69.8 *63.8*	1.42 *0.91* 69.8 *63.8*	3.11 *3.08* 76.0 *75.2*	1.64 *0.96* 76.0 *75.2*	3.27 *3.25* 72.2 *71.3*	0.97 *0.89* 72.2 *71.3*

'Profiles 50%' refers to the most frequent profiles, observed on 50% of the actual cows in herds 2

*ρ *= relative penalty reduction

## Discussion and conclusion

The stochastic optimisation method was chosen due to its simplicity: finding alternative solutions was straightforward for the issues under study, with a number of parameters (three) smaller than when using evolutionary algorithms [10]. However, except for the initial temperature where a fine-tuning method was proposed, the other parameters were held constant, possibly at suboptimal values. Furthermore, stopping rules were used to avoid excessive computation time, although a few better solutions were still found. The introduction of constraints into the SA process, (which we called the ASA process) worked correctly but certainly slowed down the convergence rate. Consequently, it cannot be claimed that the approach towards the global maximum in a given computation time is better than the evolutionary approach (this would need specific studies). It can only be noted that many local maxima of the Lagrange function were avoided and that the ultimate solution was fairly accurate for practical use. This statement was supported by the fact that running the ASA process for the Holstein breed during twice as many temperatures would have only increased the efficiency of the mating design (parameter *ρ*, see 2.4) by a very small amount: from 69.8% to 71.0%.

The two-step approach has been also used by Berg *et al. *[11], Sonesson and Meuwissen [8] and Sorensen *et al. *[12]. In our work, the main reasons for its implementation were its simplicity and the certainty that, in the first step, the general interest could be accounted for, before paying attention to private interests in the second step. However, Kinghorn and Shepherd [6] and Kinghorn *et al. *[7] have been able to implement mate selection, *i.e. *the complex combined optimisation, using evolutionary algorithms, even in the context of multi-trait selection (*i.e. *considering multiple EBV per future progeny). Here, simplicity was the primary goal, even leading us to give up the deterministic approach of [9], which also optimises mate selection. For a given computation time, we did not know whether it would be more valuable to implement the single step procedure.

The main simultaneous constraints in the mating method were that the relative penalty reductions should be the same for inbreeding and for trait defects. This might seem arbitrary. The only rationale behind this idea was that both average penalties should lie as low as possible, so as to decrease the risk of exclusion by breeders. We observed that penalties for traits could be reduced more easily than penalties for inbreeding. Thus, the effect of the design tested was assessed as a proportion of the maximal penalty reductions observed during specific optimisations. The proposed optimisation scheme required neither the population to be closed nor breeders' requests to be explicitly indicated on survey forms. The only assumption was that a given AI organization (or even breed association) willing to control inseminations in a cow population declared semen stores available for a list of chosen sires (candidates) and hopefully exceeding the insemination needs. Availability meant true availability of stores for home sires or potential availability after purchase from another AI (artificial insemination) organization if needed. Here, declared stores came from home sires and other French sires. For the Holstein breed, international sires could have been included but accessibility to their complete pedigrees could have been a limiting factor. Anyway, it was agreed that breeders were free to implement any mating of their own choice. The only ambition of the authors was to propose a high-quality and acceptable service that would be beneficial to the AI organization and increase genetic variability in the population. Conversely, if breeders were left to themselves, often with only very partial information on pedigrees, clearly they would have understandable difficulties in paying enough attention to this problem.

In the proposed optimisation scheme, some degree of disassortative mating was introduced, which normally would lead to an additional decrease of genetic variances [13]. However, it is reasonable to think that this effect would be small due to its dilution over a large number of traits (herds 1) or over a large number of combinations of traits (herds 2). For the same reasons, its effect on genetic covariances between traits may be small compared with the leading effects induced by the major selection of sires on the overall EBV, already carried out when choosing the list of candidates.

The tested optimisation method turned out to be efficient for managing both inbreeding and various requests of breeders about numerous traits. Here, optimisation concerned only one step of the dairy breeding schemes: the use of progeny-tested bulls on the commercial cow population. Other major steps, such as producing young bulls from bull sires and bull dams might be optimised along the same general principles. This should be tested in the future.

In the forthcoming years, major changes will probably occur in dairy cattle breeding schemes, as a consequence of genomic selection. In this context, very young bulls might be evaluated with high accuracy, without progeny testing [14]. Very likely, breeders will maintain and even reinforce their requests for individuals not only with high overall EBV but also with well-balanced profiles. Thus, optimising mating both for inbreeding and a multi-trait selection design will still be required. The funds saved by giving up progeny-testing should probably be partly re-invested into the marker-typing of much younger candidates than that done today, leading to what we call 'the first step' (optimising contributions before optimising matings). Accounting for balanced profiles would still be needed and the corresponding optimisation would still hold. The only major change, compared with what was carried out in the present paper, would be that higher genetic gains could be targeted in order to profit from the new potential brought by genomic selection *i.e. *instead of constraining yearly genetic gains to the observed past values, more ambitious values could be chosen. Research data not presented here for length reasons, have already shown that an efficient way to address the issue would be to introduce an extra penalty for the overall EBV and to try to reduce this penalty, along with reductions of penalties for genetic diversity and trait faults.

## Competing interests

The authors declare that they have no competing interests.

## Authors' contributions

JJC set up the methodology. KT and HdP defined breeders'requirements and prepared the corresponding files. DR processed the national files to provide the relevant information.

## Appendix 1

### Running principles of the adaptative simulated annealing

The general principles of the canonical simulated annealing are described in [15,16]. The term 'adaptative' first concerned the optimisation of a constant function, accelerated by managing the evolution of temperature and number of tested alternative solutions across steps [17]. Here, 'adaptation' should be rather compared to what is called 'Darwinian adaptative simulated annealing' [18].

Let *f*(*k*) be the function to be maximized, depending on configuration *k*. This function is penalized for *n *positive constraint functions *f*_1_(*k*)...*f*_*n*_(*k*) that should be strictly equal to 0. When optimising sire contributions, *f*(*k*) = $-\bar{\varphi }(k)$ and $f_{1}(k)={\left(\bar{W}(k)-\tilde{W}\right)}^{2}$.

When optimising matings, $f(k)=\bar{\rho }(k)$, *f*_1_(*k*) = (*ρ*_*F*_(*k*) - *ρ*_*T*_(*k*))^2 ^and *f*_2_(*k*) = (1 - *C*(*k*)/$\tilde{C}$)^2^. For the meaning of symbols, see the main text. The corresponding Lagrange function is $H(k)=f(k)-\sum_{i=1}^{i=n}\lambda_{i}f_{i}(k)$, where the *λ*_*i *_are Lagrange multipliers. These coefficients were calculated adaptatively at the end of each step, when all the permutations pertaining to a given temperature were finished. For each permutation, accepted or not, variations of functions *δf*, *δf*_1_,... *δf*_*n *_were observed, with standard deviations *σ*, *σ*_1_,..., *σ*_*n*_. The standardized variations are *δ*_*f*_/*σ*, *δf*_1_/*σ*_1_,..., *δf*_*n*_/*σ*_*n*_. For these variations, the vector of Lagrange multipliers is ***ω ***= *σ *(*λ*_1_/*σ*_1_...*λ*_*n*_/*σ*_*n*_)' and the weights for δH*(*k*) on the standardized scales are $\left(\begin{array}{c} 1\\ \omega \end{array}\right)$. Let **r **be the vector of correlations between *δf *and the other *δ*. Let **C **be the correlation matrix of size *n *between the penalizing *δ*. The vector ***ω ***is such that the covariances between δH* and the penalized *δ*. have the same negative value: *α*. Then, ***ω***_*α *_= **C**^-1^(**r **- *α***1**_*n*_) = **C**^-1^**s**_*α *_and $\sigma^{2}(\delta H^{*})=1+2r^{\prime }s_{\alpha }+{s^{\prime }}_{\alpha }s_{\alpha }$. Consequently, the correlation between *δH *and *δf *is $R_{1}=\frac{1-{\omega^{\prime }}_{\alpha }r}{\sigma (\delta H^{*})}$ and the correlations between *δH *and the other *δ*. are all equal to *R*_2 _= *α*/*σ *(δH*). Finally, *α *was chosen so that the difference *R*_1 _- *R*_2 _would be maximal ('adaptation' to increase the main function and to decrease the penalizing functions) and obtaining the value of the optimal ***λ***, to be used in the next step, was straightforward. This could be carried out by the Newton-Raphson method. Although we never observed that this optimisation would return an undesirable negative value for *R*_1_, we preferred to use a grid search and to check for desirability. The general effect of the method was that constraints were very tightly fast fulfilled and that the main function increased steadily over time.

## Appendix 2

### Managing temperature in simulated annealing

The only fine-tuning concerned the initial temperature *θ*_1_. Let *x *be the variation of the function of interest after one permutation (practically, we considered the leading function without constraints). The overall distribution of *x *is considered to be *N*(*μ*, *σ*^2^). The running rule of the simulated annealing for maximizing the function is to accept the permutations when *x *is positive or 0 and otherwise with a probability exp[*x*/*θ*_*t*_] where *θ*_*t *_is temperature at step *t*. Then, the initial overall acceptance rate is $\alpha =\Phi (\mu /\sigma )+\int_{-\infty }^{0}\exp[x/\theta_{1}]\phi (\frac{x-\mu }{\sigma })\frac{dx}{\sigma }$ where *ϕ*, Φ are respectively the probability density and the distribution function of the standard Gaussian distribution. The integral is equal to $\exp[\frac{\mu }{\theta_{1}}+\frac{\sigma^{2}}{2\theta_{1}^{2}}]\Phi (-\frac{\mu }{\sigma }-\frac{\sigma }{\theta_{1}})$. In a burn-in run, *μ *is very close to 0 and the expression of the acceptance rate becomes quite simple: $\alpha =1/2+\exp[\frac{\sigma^{2}}{2\theta_{1}^{2}}]\Phi (-\frac{\sigma }{\theta_{1}})$. *θ*_1 _corresponding to a desired *α *can be easily calculated by a Newton-Raphson procedure. It turned out that *θ*_1 _is of the same magnitude order as *σ*. For instance, *θ*_1 _= 0.5*σ *for *α *= 2/3 and *θ*_1 _= 1.25*σ *for *α *= 0.8. The initial temperature was calculated so that the acceptance rate would be equal to 0.80, a trade-off value for avoiding two major risks: either losing time with a very slow progress of solutions or a very fast progress towards a local minimum. Thus, initially, 60% of 'bad' permutations were accepted.

Otherwise, simple rules were used. First, the rate of temperature decrease was constant and very slow (*θ*_*t*+1 _= 0.99*θ*_*t*_) in order to avoid being trapped in local maxima and second, the number of alternatives at a given temperature was constant (equal to either the number of available doses or the number of cows, according to the case).

## Appendix 3

### Finding the optimal contributions of sires

Let column vector **n **of size *s *be the vector of the numbers of cows allocated to each of the *s *sires, given that $1_{s}^{'}n=M$, the overall number of cows. Their relationship matrix is **A **and the column vector of their average relationships with the cow population is **p**. It has already been shown [9] that the average coancestry coefficient $\bar{\varphi }$ in the existing female population augmented by the future females to be born from matings (resulting in **n**) is equal to a constant (not depending on **n**) + a quadratic form depending on **n **and proportional to function 0.5**n'An **+ **p'n**. Then, the leading function *f *for the adaptative simulated annealing is -0.5**n 'An **- **p'n**. Let column vector **w **be the vector of the overall EBV of sires. Then, the penalizing function is $f_{1}={\left(\bar{W}-\tilde{W}\right)}^{2}$ where $\bar{W}=\frac{w'n}{M}$ and $\tilde{W}$ is the desired value.

Constraints for available doses were not introduced as additional functions because they were met by any alternative solution during the annealing process. First, the numbers of available doses were transformed into integer numbers of cows after considering the average number of doses needed per gestation. The corresponding column vector is **d **of sum D, the overall number of transformed doses. Vector **u **of size D indicates (1 or 0) which doses were used. This vector was set to 0 before starting the ASA process. Vector **z **of size D gives the identification of the corresponding sires.

To provide an initial solution, M 1's were randomly allocated to M addresses in vector **u **and the corresponding vector **n **was calculated. To provide an alternative solution, 2 integers *i *and *j *were drawn randomly in the interval [1 D] until *d*_*i *_= 1 and *d*_*j *_= 0 (or the reverse) and then, the alternative solution was obtained from swapping (*d*_*i *_= 0 and *d*_*j *_= 1), which modified vector *n*, after considering sire identifications *z*_*i *_and *z*_*j *_(*n*(*z*_*i*_) was decreased by 1 and *n*(*z*_*j*_) was increased by 1). Computing the variations of functions *f *and *f*_1 _induced by swapping was straightforward.

## Appendix 4

### The lists of traits considered

Thresholds or pairs of threshold values considered for the EBV of progeny are indicated in brackets.

Holstein breed (21 type traits, seven functional traits, five production traits)

Type traits: angularity, body depth, body capacity (0), chest width, foot angle, final score, fore teat placement, fore udder, height at sacrum (0), locomotion (0), rear legs set, rear legs rear view, rear teat placement, rear udder height, rump angle (optimum between 0 and 1), teat length, udder (0.5), udder balance (optimum between 0 and 1), udder depth, udder support, width at pins.

Functional traits: cell count (0.2), ease of birth, ease of calving, fertility (0), functional longevity, milking speed (-1), temperament.

Production traits: fat content (-1.5), INEL, ISU, milk yield (+300), protein content (0).

Normandy breed (19 type traits, six functional type traits, five production traits)

Type traits: chest depth, chest width, feet and legs (-0.5), final score, fore teat placement (-0.5), fore udder, frame, height at sacrum (-0.5), muscularity, rear legs set, rear udder height, rump angle, rump length, teat direction, udder, udder balance (-0.5), udder depth (0), udder support (0), width at pins (-0.5).

Functional traits: cell count (-0.5), ease of birth, ease of calving, fertility (-0.5), functional longevity, milking speed (-0.5).

Production traits: fat content (-0.5), INEL, ISU, milk yield (+250), protein content.
